# Correcting depressed distal radial malunions at distal levels via a 5 mm incision

**DOI:** 10.3389/fsurg.2026.1825806

**Published:** 2026-07-08

**Authors:** Hao Yao, Jingqiao Li, Xu Zhang, Bin Wang, Xuewu Zhou, Xiaoliang Yang

**Affiliations:** 1 The First Department of Orthopaedic and Department Surgery, Yichang Central People's Hospital, the First College of Clinical Medical Science, China Three Gorges University, Yichang, Hubei, China; 2 Orthopaedic Surgery, West Medical Center of Shijiazhuang, Jingxing Hospital, Shijiazhuang, Hebei, China; 3 Department of Hand Surgery, Third Hospital of Hebei Medical University, Shijiazhuang, Hebei, China

**Keywords:** 5 mm incision, bone grafting, bone healing, depressed distal radial malunion, PIN

## Abstract

**Objective:**

This study aims to evaluate the efficiency of correcting depressed distal radial malunions at distal levels via a 5 mm incision.

**Methods:**

Between May 2021 and October 2023, 46 patients (46 wrists; external fixation group) with depressed distal radial malunions at the distal levels were treated. For comparison, we reviewed another 49 patients (49 wrists; plating group) treated with conventional open reduction and internal fixation with plate and crew systems in an earlier period.

**Results:**

In the plating group, secondary surgery to remove the plate was performed in 32 of 49 patients. There were significant differences in off work time (152 ± 42 vs. 210 ± 72; *P* < 0.01) and total treatment cost and loss due to off work (US$ 32,653 ± 1,234 vs. US$ 43,653 ± 3,234; *P* < 0.01). There were significant between-group differences in radial height (12.1 ± 3.9 mm vs. 10.2 ± 2.7 mm; *P* < 0.05) and ulnar variance (0.5 ± 0.2 mm vs. 1.7 ± 1.2 mm; *P* < 0.05). There was no significant difference in the Mayo Wrist Scores (94.3 ± 5.7 vs. 92.8 ± 7.1; *P* > 0.05), including excellent (32 vs. 33), good (14 vs. 13), and fair (0 vs. 3) results. The patient aesthetics scores were 8.8 ± 1.8 vs. 8.1 ± 1.1 (*P* < 0.05). The patient satisfaction scores were 8.9 ± 1.6 vs. 8.1 ± 1.2 (*P* < 0.05) based on the Short Assessment of Patient Satisfaction questionnaire.

**Conclusions:**

Depressed distal radial malunions at the distal levels can be treated via a 5 mm incision. External fixation through this small incision may be effective in maintaining radial height, especially in patients with osteoporosis. Usually, owing to a non-necessity for secondary implant removal in some patients, this technique results in acceptable outcomes in total treatment cost, total off work time, and patient satisfaction. The technique can be an alternative in addition to plate fixation. However, more factors should be considered when selecting the treatment strategy.

## Introduction

Distal radius fractures account for up to 17% of all extremity fractures (1). Depressed distal radius malunion (DDRM) is a major complication, with an overall reported incidence of 33%, especially in patients treated conservatively (2). Open osteotomy and plate fixation are the most used techniques to restore normal alignment (3). However, more than 30% of patients require plate removal at a later stage because plates positioned distally can protrude and irritate the tendons due to plate prominence (4).

When correcting DDRM, if the distal radius fracture is located at or distal to the distal margin of the pronator quadratus, the plate should be placed more distally, which can lead to plate prominence (5). Based on the Soong classification, plate prominence is divided into grade 0 (the plate is dorsal to the volar critical line and proximal to the volar rim), grade 1 (the plate is palmar to the volar critical line but proximal to the volar rim), and grade 2 (the plate extends beyond the level of the volar rim) (6). Grade 2 is associated with a significant need for implant removal due to flexor tenosynovitis (7–10). To avoid this plate irritation, we fix the fracture with external fixators. Moreover, Akar et al. (11) suggested that closed reduction and external fixation is effective in achieving successful treatment outcomes because of the effect of ligamentotaxis. They showed that an optimal radioulnar angle is critical for clinical and functional success.

This retrospective study aimed to evaluate the efficiency of minimally invasive fixation for the treatment of DDRMs at the distal levels, followed by external fixation and bone grafting through a small incision. The study hypothesis was that the technique can achieve acceptable reduction without causing significant complications. We also compared it with another group of patients who underwent conventional open reduction and plate fixation in an earlier period.

## Materials and methods

This study was performed in accordance with the Declaration of Helsinki. Third Hospital of Hebei Medical University. Informed consent was obtained from each patient.

**Figure 1:**
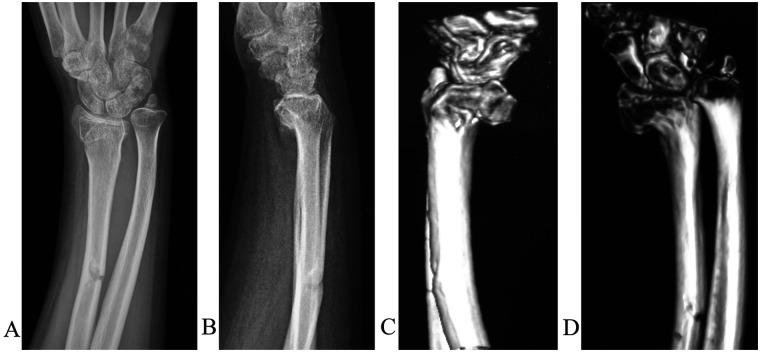
A 36-year-old female patient suffered from depressed distal radial malunion (initial type B3) because of a fracture that occurred 4 months before hospital admission. **(A)** A posteroanterior (PA) X-ray. **(B)** A lateral X-ray. **(C)** A radial view of a three-dimensional CT scan. **(D)** An anteroulnar view.

Between May 2021 and October 2023, consecutive patients with DDRMs at or distal to the distal margin of the pronator quadratus were selected based on the eligibility criteria as follows: (1) patients aged between 18 and 65 years; (2) a confirmed diagnosis of distal radius malunion with callus bridging of the fracture line; (3) an inclination of ≤15°, a dorsal tilt of ≥10°, or an ulnar variance of ≥3 mm (depressed fracture) (5); (4) original AO/OTA (AO Foundation and Orthopaedic Trauma Association) Classification type A2 and A3 fractures; (5) original-type C2 and C3 fractures; (6) extra-articular fractures or intra-articular fractures with congruence of the articular surface; and (7) the fracture level distal to the distal margin of the pronator quadratus (the level of 3 cm proximal to the wrist) (5). Patients <18 years were excluded because the malunion could be corrected with growth. We excluded DDRMs located proximally to the distal margin of the pronator quadratus. We excluded C1- and C2-type fractures with displacement of the articular surface because we found that reduction through a small incision was difficult (*n* = 4). We excluded type A1 fractures because they did not involve the distal radius (*n* = 2). We excluded type B2, B3, and C3 fractures because of a complex procedure for a minimally invasive technique (*n* = 2). Patients who declined to undergo the surgery were excluded. Other exclusion criteria included combined triangular fibrocartilage complex injuries, pathologic fractures, uncooperative adults, infection, diabetes, rheumatoid arthritis, or gout. The osteotomy plane was confirmed based on preoperative X-rays and CT images (Figures 1A–D). Patients were allocated by the same senior orthopedic surgeon (XY) based on those criteria. The same surgical team performed all operations.

### Surgical technique

**Figure 2:**
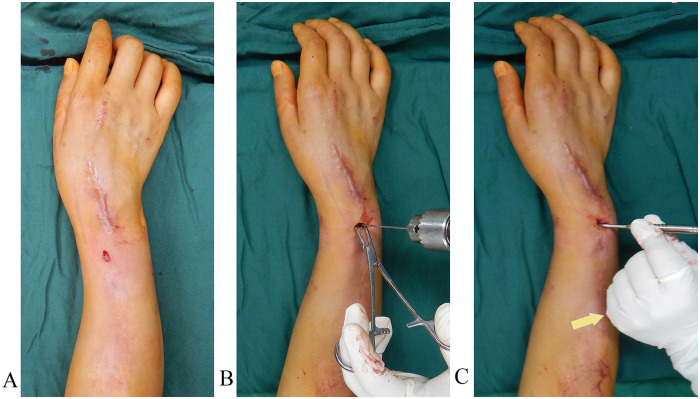
**(A)** A 5 mm incision between the extensor pollicis longus and the extensor digitorum communis, 5–10 mm distal to the osteotomy level (avoid skin laceration during late distraction). **(B)** Multiple pin holes were created using a 1.0–1.2 mm K-wire in different directions in the osteotomy plane. **(C)** Osteotomy was performed by hammering a small seating chisel. The chisel was supported with the hand and forearm (arrow), avoiding deep insertion, which may injure the tendons and nerves.

**Figure 3:**
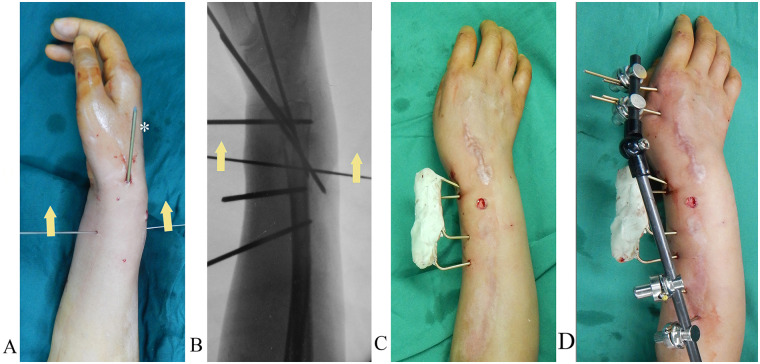
**(A)** A 2.5 mm pin was inserted manually, 5–10 mm distal to the fracture site (avoiding late skin laceration during distraction), and slid into the fracture site. The pin was continuously passed through the fracture site and it exerted pressure on the dorsal skin incision. By pulling both ends of the pin distally (arrows), the distal bone fragment was distracted to restore the normal length of the radius and meanwhile to restore volar tilt and radial inclination. To maintain the reduction, one or two 3.0 mm pins (*) were provisionally introduced, obliquely in a distal-to-proximal direction. **(B)** A bony distraction maneuver on X-ray. **(C)** Two to three 3.0 mm pins were inserted in the distal fragment and two pins in the proximal fragment. The pin ends were bent toward the fracture site about 2 cm away from the skin. Bone cement was mounted on the pin ends. **(D)** A bridging external fixator was applied against axial load.

The operation was performed under general or brachial plexus anesthesia with tourniquet control. We marked the osteotomy level using fluoroscopy. We made a 5 mm incision between the extensor pollicis longus and the extensor digitorum communis, 1 cm distal to the osteotomy level (avoiding late skin laceration during distraction) (Figure 2A). For malunions longer than 3 months, we created multiple holes in different directions using a 1.0–1.2 mm K-wire through this small incision (Figure 2B). We performed osteotomy by hammering a small seating chisel. During this procedure, care was taken to support the chisel with the hand and forearm, avoiding deep insertion, which may injure the tendons and nerves (Figure 3C). For malunions equal or less than 3 months, osteotomy could be performed manually with a small chisel or bone cutter because the fibrocartilaginous callus was soft. Then, we rotated the chisel to fracture the radius. A 2.5 mm pin was inserted manually, 1 mm distal to the fracture site (avoiding skin laceration during distraction), and slid into the fracture site. The pin was continuously passed through the fracture site and came out of the dorsal skin. By pulling both ends of the pin distally, we distracted the distal bone fragment to restore radial height and meanwhile to restore volar tilt and radial inclination. To maintain the reduction, one or two 3.0 mm Steinmann pins were provisionally introduced, obliquely in a distal-to-proximal direction (Figures 3A,B). Satisfactory reduction was confirmed using fluoroscopy. Then, we introduced one or two 3.0 mm transverse bicortical pins into the proximal fragment and kept two to three 3.0 mm pins in the distal fragment. Satisfactory reduction and pin position were confirmed using fluoroscopy. The pins were bent toward the fracture site about 2 cm away from the skin. We mixed monomer (liquid) and polymer (powder) of bone cement (Single dose 40 g; PALACOS®, Hanau, Germany). When the viscosity of the bone cement changed over time from a runny liquid into a dough-like state, we applied it to the bent pin ends and waited for it to harden into a solid material. Thus, we created a frame to prevent pin migration. Satisfactory reduction and pin position were confirmed using fluoroscopy. The void at the fracture site was filled with iliac crest autografts through a 4.5 mm drill guide at the small incision. The wrist was immobilized using a joint-spanning external fixator (four 3.5 Schanz pins; Shuangyang Medical Instrument Co., Ltd. Zhangjiagang City, China) to reinforce maintenance of radial height (Figures 3C,D).

**Figure 4:**
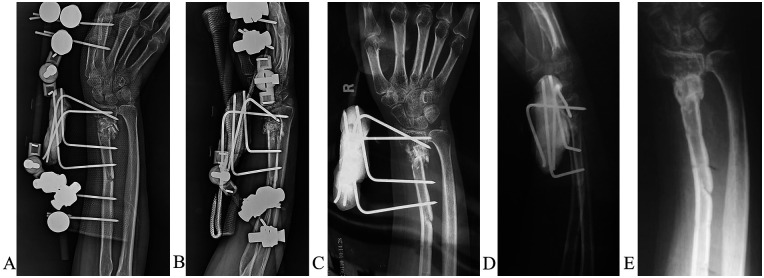
**(A)** A PA X-ray taken immediately after surgery. **(B)** A lateral X-ray. **(C)** Bone healing 1.5 months after surgery on an anteroposterior X-ray. **(D)** A lateral X-ray. **(E)** A PA X-ray taken after 2 years.

After surgery, X-rays were taken every 2 weeks until bone healing occurred (Figures 4A–C). The cemented pin frame was protected using a dorsal short-arm splint or cast. The external fixator was removed 6 weeks after surgery, which allowed wrist movement. After bone healing, the pines were cut off and the frame and pins were removed.

### Outcome evaluation

We assessed the palmar tilt of the radius on lateral X-rays and radial inclination, height, and ulnar variance on posteroanterior X-rays (12) (Figure 4D). We assessed pin site infection based on clinical symptoms (13). We assessed wrist pain intensity using the visual analog scale (VAS) (14). The active range of motion of the wrist was measured using a goniometer (15). All measurements were compared with those on the opposite side. Grip strength of the hand was assessed using a baseline hydraulic hand dynamometer (Fabrication Enterprises Inc., White Plains, NY, USA) (16). We assessed the pronation torque using the McConkey method at 5 positions of rotation (90° of supination, 45° of supination, neutral, 45° of pronation, and 80° of pronation) (17). To remove any discrepancy between dominant and non-dominant hand strength, we based the scores for analysis on the premise that the grip strength was 15% higher at the dominant side than at the non-dominant side; and no correction was required for left-handed patients (18). We rated wrist pain and hand numbness of the patients using a 10 cm visual analogue scale (19). We used the Mayo Wrist Score to assess wrist function (90–100, excellent; 80–89, good; 60–79, satisfactory; <60, poor) (20). Aesthetics and patient satisfaction were assessed using the 10 cm visual analog scale (21).

### Statistical analysis

Continuous variables were expressed as mean ± standard deviation. Normality was assessed using the Shapiro–Wilk test. A paired *t*-test was used to compare the radiological parameters within each group. Pearson's chi-square test was used to compare categorical variables. An independent *t*-test was used to compare the parameters between the two groups. A value of *P* < 0.05 was considered statistically significant. The collected data were analyzed using the Statistical Package for Social Sciences 20.0 (SPSS, Inc., Chicago, IL, USA).

## Results

We selected 46 patients (external fixation group; 46 upper limbs) from 52 patients based on the inclusion and exclusion criteria. For comparison, we reviewed another 49 patients (plating group; 49 wrists; control group) who were treated with open reduction and internal fixation with plate and crew systems from January 2019 to April 2021. The patients were also selected based on the same inclusion and exclusion criteria (Table 1). One year after surgery, secondary surgery to remove the plate was performed in 32 of the 49 patients.

**Table 1:** Preoperative demographics for 46 patients with depressed distal radial malunion.

Item	External fixation	Plating	*t*	*P*-value
	(*n* = 46)	(*n* = 49)		
Age (year, range)	35.3 ± 14.7 (19–52)	36.2 ± 15.2 (18–55)	−0.31	0.76
Sex (m:f)	20:26	21:28	−3	0.21
Body mass index	31.4 ± 6.2	32.1 ± 6.7	−0.53	0.09
Smoking (*n*)	5	7	−3	0.25
Side (l: r)	21:25	24:25	−1	0.5
Dominant hand (*n*)	27	29	−3	0.21
Cause (*n*)				
Fall	29	26	−0.65	0.55
Road traffic accident	10	12		
Sports	3	4		
Machinery	2	4		
Others	2	3		
Duration from injury to surgery (month)	6.7 ± 3.8	6.1 ± 3.7	0.77	0.45
Intraarticular fracture (*n*)	4	6	−3	0.21
Depression (mm)	6.3 ± 2.5	6.1 ± 3.4	0.33	0.74
Osteoporosis (*n*)	15	17	−1.2	0.26
Preoperative X-ray parameter				
Loss of radial height (mm)	6.2 ± 2.6	5.9 ± 2.9	0.58	0.56
Palmar tilt (°)	27.3 ± 5.5	26.9 ± 5.8	0.35	0.73
Radial inclination (°)	15.2 ± 4.5	14.8 ± 5.1	0.41	0.68
Ulnar variance (mm)	4.2 ± 1.8	4.7 ± 1.5	−1.52	0.13
Original AO/OTA (*n*)				
A2	22	21	−0.73	0.52
A3	13	15		
C1	9	8		
C2	2	5		
Operative time (minute)	53 ± 23	77 ± 36	−3.85	<0.01
Blood loss (mL)	42 ± 23	63 ± 32	3.69	<0.01
Infection (*n*)				
Deep abscess	0	0		
Cellulitis	0	1		
Pin site	2			
Donor site	0	0		
Bone healing (week)	6.3 ± 2.3	6.5 ± 2.5		
Plate removal (*n*)		32		
Off work time (day)	152 ± 42	210 ± 72	−5.68	<0.01
Total cost and loss (US$)	32,653 ± 1,234	43,653 ± 3,234	−18.97	<0.01
Final follow-up (month)	28.3 ± 3.2	27.7 ± 3.8	0.83	0.41

Data are shown as mean ± standard deviation; DEF, dual external fixation; plate fixation; AO/OTA, AO Foundation and Orthopaedic Trauma Association.

The mean age of the external fixation group and plating group was 35.3 ± 14.7 years (range, 19–52 years) vs. 36.2 ± 15.2 years (range, 18–55 years), respectively (95% CI: −5.1, 3.3; df = 93; *P* = 0.76) (Table 1). The time intervals between injury and surgery were 6.7 ± 3.8 and 6.1 ± 3.7 months (95% CI: −0.85, 2.05; df = 93; *P* = 0.45). There were significant differences in off work time (152 ± 42 vs. 210 ± 72; 95% CI: −12,345.6, −9,854.4; df = 93; *P* < 0.01) and total treatment cost and loss due to off work (US$ 32,653 ± 1,234 vs. US$ 43,653 ± 3,234; 95% CI: −12,345.6, −9,854.4; df = 93; *P* < 0.01).

We found no significant differences in preoperative radiographic loss of radial height (6.2 ± 2.6 mm vs. 5.9 ± 2.9 mm; 95% CI: −1.52, 2.32; df = 93; *P* = 0.56), palmar tilt (27.3° ± 5.5° mm vs. 26.9° ± 5.8° mm; 95% CI: −0.85, 2.05; df = 93; *P* = 0.73), radial inclination (15.2° ± 4.5° vs. 14.8° ± 5.1°; 95% CI: −1.1, 1.9; df = 93; *P* = 0.68), or ulnar variance (4.2 ± 1.8 mm vs. 4.7 ± 1.5 mm; 95% CI: −1.15, 0.15; df = 91; *P* = 0.13). There were significant differences in operative time (53 ± 23 min vs. 77 ± 36 min; 95% CI: −1.15, 0.15; df = 93; *P* < 0.01) and blood loss (42 ± 23 mL vs. 63 ± 32 mL; 95% CI: −0.83, 2.03; df = 92; *P* < 0.01).

We measured radiographic parameters immediately after surgery (Table 2). We found no significant between-group differences in radial height (42 ± 23 mm vs. 63 ± 32 mm; 95% CI: −0.85, 3.45; df = 52.3; *P* = 0.16), palmar tilt (15.6° ± 3.8° vs. 16.4° ± 3.3°; 95% CI: −2.15, 0.55; df = 93; *P* = 0.31), radial inclination (42° ± 23° vs. 63° ± 32°; 95% CI: −2.01, 1.61; df = 93; *P* = 0.83), or step off (42 ± 23 mm vs. 63 ± 32 mm; 95% CI: −0.21, 0.01; df = 93; *P* = 0.16). We found a significant difference in ulnar variance (0.4 ± 0.2 mm vs. 0.5 ± 0.3 mm; 95% CI: −0.199, −0.001; df = 89.3; *P* = 0.04).

**Table 2:** Radiological parameters collected immediately after surgery versus those collected at the final follow-up.

Item	External fixation	Plating	*t*	*P*-value
	(*n* = 46)	(*n* = 49)		
Radial height (mm)				
IAF	12.3 ± 1.1	11 ± 5.6	1.42	0.16
FF	12.1 ± 3.9	10.2 ± 2.7	2.85	<0.01
*t*	0.32	0.89		
*P*-value	0.75	0.37		
Palmar tilt (°)				
IAF	15.6 ± 3.8	16.4 ± 3.3	−1.02	0.31
FF	15.3 ± 3.7	16.3 ± 3.6	−1.34	0.19
*t*	0.39	0.14		
*P*-value	0.7	0.89		
Radial inclination (°)				
IAF	23.5 ± 4.3	23.7 ± 4.6	−0.22	0.83
FF	22.4 ± 4.6	22.8 ± 4.3	0.45	0.65
*t*	1.2	0.98		
*P*-value	0.23	0.33		
Ulnar variance (mm)				
IAF	0.4 ± 0.3	0.5 ± 0.3	−2.05	0.04
FF	0.5 ± 0.4	1.7 ± 1.2	−6.85	<0.01
*t*	1.38	6.78		
*P*-value	0.17	<0.01		

Data are shown as mean ± standard deviation. IAS, immediately after surgery; FF, final follow-up.

We measured radiographic parameters at the final follow-up. We found significant between-group differences in radial height (12.1 ± 3.9 mm vs. 10.2 ± 2.7 mm; 95% CI: 0.52, 3.28; df = 93; *P* < 0.01) and ulnar variance (0.5 ± 0.2 mm vs. 1.7 ± 1.2 mm; 95% CI: −1.54, −0.86; df = 93; *P* < 0.01). We found no significant differences in palmar tilt (15.3° ± 3.7° vs. 16.3° ± 3.6°; 95% CI: −2.49, 0.49; df = 92; *P* = 0.19) or radial inclination (22.4 ± 4.6 vs. 22.8 ± 4.3°; 95% CI: −1.75, 0.95; df = 93; *P* = 0.65).

In the external fixation group, we found no significant differences between the radiographic parameters measured immediately after surgery and those at the final follow-up: radial height (12.3 ± 1.1 mm vs. 12.1 ± 3.9 mm; 95% CI: −0.85, 1.25; df = 93; *P* = 0.75), palmar tilt (15.6° ± 3.8° vs. 15.3° ± 3.7°; 95% CI: −1.228, 1.828; df = 93; *P* = 0.7), radial inclination (23.5° ± 4.3° vs. 22.4° ± 4.6°; 95% CI: −0.82, 2.02; df = 93; *P* = 0.23), or ulnar variance (0.4 ± 0.3 mm vs. 0.5 ± 0.4 mm; 95% CI: −0.82, 2.02; df = 92.3; *P* = 0.17).

In the plating group, we found significant differences between the radiographic parameters measured immediately after surgery and those at the final follow-up: ulnar variance (0.5 ± 0.3 mm vs. 1.7 ± 1.2 mm; 95% CI: 0.845, 1.555; df = 54; *P* < 0.01). We found no significant differences in radial height (11 ± 5.6 mm vs. 10.2 ± 2.7 mm; 95% CI: −0.85, 2.25; df = 93; *P* = 0.37), palmar tilt (16.4° ± 3.3° vs. 16.3° ± 3.6°; 95% CI: −1.02, 1.22; df = 93; *P* = 0.89), or radial inclination (23.5° ± 4.3° vs. 22.4° ± 4.6°; 95% CI:−0.715, 2.915; df = 93; *P* = 0.33).

**Figure 5:**
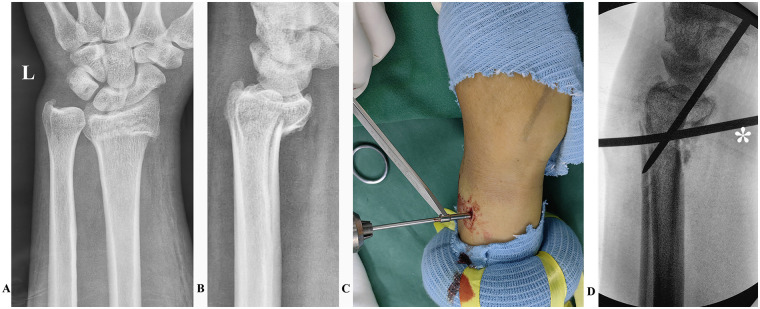
A 51-year-old male patient suffered from depressed distal radius fracture (initial type C2) 4 months before admission. **(A)** A PA X-ray taken immediately after surgery. **(B)** A lateral X-ray. **(C)** Multiple pin holes were made for osteotomy. **(D)** The distal bone fragment was distracted with a pin (*) to restore the normal length of the radius.

**Figure 6:**
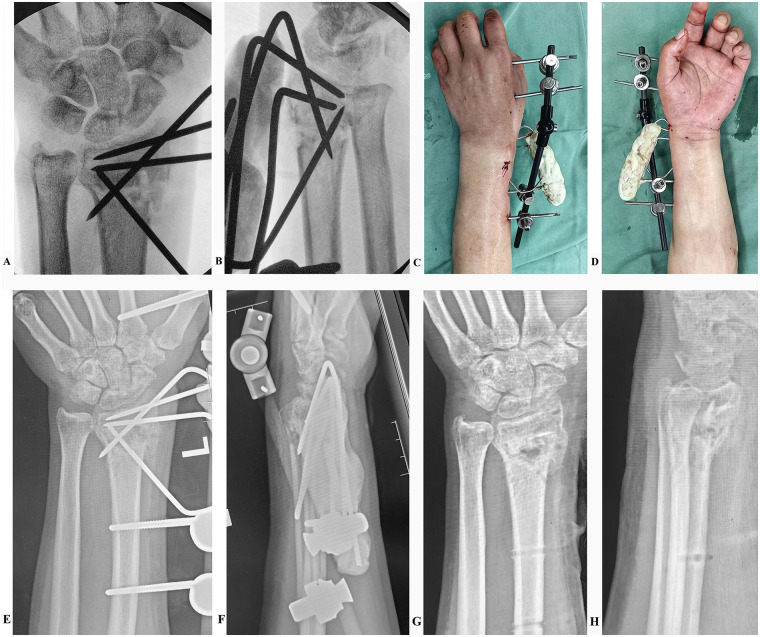
Immediately after surgery. **(A)** A PA X-ray. **(B)** A lateral X-ray. **(C)** A dorsal appearance of fixation. **(D)** An anterior appearance of fixation. **(E)** Bone healing shown on a PA X-ray after 6 weeks. **(F)** A lateral X-ray. **(G)** A PA X-ray taken 2 months after surgery. **(H)** A lateral X-ray.

**Figure 7:**
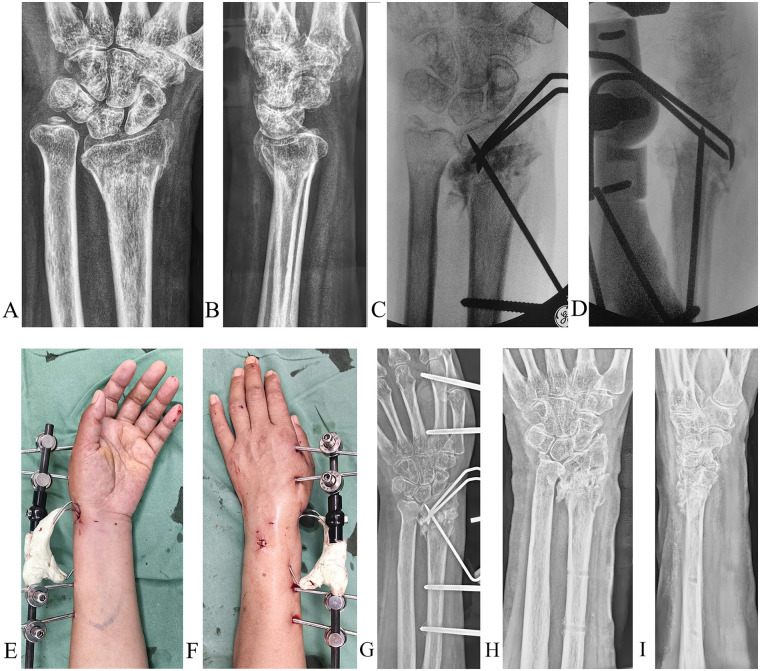
A 37-year-old female patient suffered from depressed distal radial malunion (initial type C3) because of a fracture that occurred 6 months before admission. **(A)** A preoperative PA X-ray. **(B)** A lateral X-ray. **(C)** A PA X-ray taken immediately after correction and bong grafting. **(D)** A photograph showing the anterior aspect of the injured forearm. **(E)** A photograph showing the posterior aspect of the forearm. **(G)** An AP X-ray taken 6 weeks after surgery. **(H)** A lateral X-ray.

**Figure 8:**
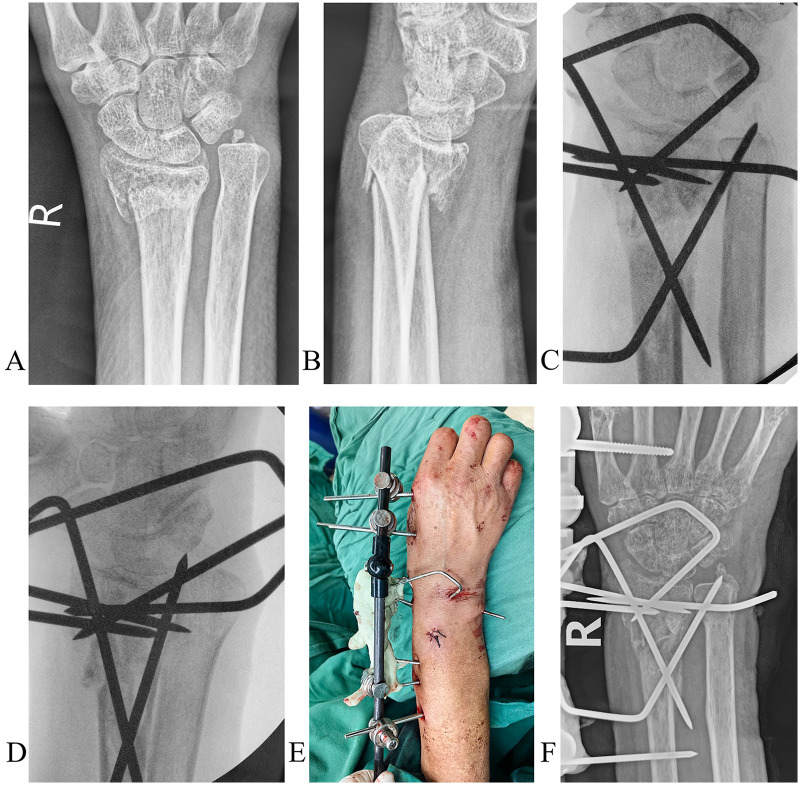
A 58-year-old female patient suffered depressed distal radius malunion (initial type A2) 3 months after fracture. **(A)** A PA X-ray. **(B)** A lateral X-ray. **(C)** A PA X-ray taken immediately after surgery. **(D)** An oblique X-ray. **(E)** A photograph showing the dorsal aspect of the forearm. **(F)** A PA X-ray taken 2 months after correction.

At the final follow-up (28.3 ± 3.2 vs. 27.7 ± 3.8; *P* = 0.41), active wrist movement and hand grip strength were similar in the two cohorts (Table 3). We found no significant differences in the Mayo Wrist Scores (94.3 ± 5.7 vs. 92.8 ± 7.1; *P* = 0.26), including excellent (32 vs. 33), good (14 vs. 13), and fair (0 vs. 3) results. The patient aesthetics scores were 8.8 ± 1.8 vs. 8.1 ± 1.1 (*P* = 0.04). The patient satisfaction scores were 8.9 ± 1.6 vs. 8.1 ± 1.2 (*P* < 0.01). There was a simultaneous increase in radial height restoration, grip strength, and wrist function (r = 1). Figures 5–8 show malunions of type C2, C3, and A2 original fractures.

**Table 3:** Outcomes at the final follow-up.

Item	External fixation	Plating	*t*	*P*-value
	(*n* = 46)	(*n* = 49)		
Active ROM (°)				
Flexion	72 ± 4	70 ± 7	1.68	0.1
Extension	62 ± 5	64 ± 6	−1.85	0.07
Radial deviation	24 ± 8	22 ± 6	1.42	0.16
Ulnar deviation	17 ± 7	16 ± 6	0.78	0.44
Pronation	74 ± 15	76 ± 17	−0.61	0.54
Supination	81 ± 10	83 ± 12	−0.89	0.38
Grip strength (%)^a^	95 ± 3	93 ± 6	2.07	0.06
Supination torque (%)^b^				
90° of supination	89 ± 7	92 ± 9	−1.85	0.07
45° of supination	93 ± 7	91 ± 8	1.34	0.18
Neutral	92 ± 3	93 ± 6	1.04	0.3
45° of pronation	93 ± 5	95 ± 6	−1.85	0.07
80° of pronation	95 ± 5	93 ± 6	1.77	0.08
Complication				
Wrist pain (MWS)	1.12 ± 0.32	1.9 ± 0.57	−7.85	<0.01
Hand numbness (VAS, cm)	1.05 ± 0.72	1.14 ± 0.86	−0.55	0.58
Tendon rupture (n)	0	0		
Carpal tunnel syndrome (*n*)	0	0		
Mayo Wrist Score	94.3 ± 5.7	92.8 ± 7.1	1.14	0.26
Excellent (*n*)	32	33		
Good (*n*)	14	13		
Fair	0	3		
Poor	0	0		
Aesthetics (VAS, cm)	8.8 ± 1.8	8.1 ± 1.1	2.05	0.04
Satisfaction (VAS, cm)	8.9 ± 1.6	8.1 ± 1.2	2.75	<0.01

Data are shown as mean ± standard deviation; ROM, range of motion; VAS, visual analog scale.

a Fifteen percent higher at dominant sides compared with the non-dominant side discrepancy, in which percentages show involved limb compared with the opposite normal side.

b Supination torque based on the McConkey method, compared with the opposite side as percentages.

## Discussion

We found that DDRMs at the distal levels could be treated through a small incision, followed by external fixation, especially for original AO/OTA Classification type A2 and A3 fractures and type C1 and C2 fractures without the need for correcting intraarticular step off. It may be more effective than plate fixation in maintaining radial height. Usually, owing to a non-necessity for secondary implant removal in some patients, the technique results in overall less total treatment cost, less total off work time, and better patient satisfaction. This technique can be an alternative in addition to plate fixation.

DDRMs affect wrist biomechanics and can result in pain, limited motion, and functional limitations (3, 4). Ali et al. (5) defined DDRMs as a radial inclination of ≤15°, a dorsal tilt of ≥10°, or an ulnar variance of ≥3 mm. They found that patients (aged between 18 and 65 years) who sustain DDRMs are more likely to have adverse activity limitations and pain, especially the loss of radial height. Although the degree of clinical severity does not correlate with bony deformity, patients with symptomatic DDRMs can experience high disability. DDRMs with dorsal tilt > 10°, radial shortening > 3 mm, or intra-articular displacement > 2 mm indicate the need for a discussion regarding the benefits of surgical correction (6, 7, 22). Currently, there are no absolute surgical indications for DDRM correction. Consideration should include patient age, wrist pain, grip weakness, loss of mobility, and possibility of infection. The conventional surgery is performed through a 5- to 10-cm volar approach (8). After osteotomy, the fixation is achieved with a volar plate and screw system. This approach allows good visual approximation of the volar cortex, allowing the lift maneuver to aid in reduction. However, structural bone grafting is often needed to reduce the risk of non-union and possible supporting failure (9). Owing to the increased ulnar variance after osteotomy, the procedure may be combined with an ulnar head resection. Moreover, bone grafts are often needed for proper reshaping. Graft loosening, migration, and absorption, as well as poor bone quality, may lead to supporting failure, redisplacement, and unpredictable outcomes (10, 11). Further surgery for removal of implant may be required in some patients, followed by at least a 6-month period of non-performance of manual work. Magtoto et al. (23) corrected the distal radius malunion using sliding volar hinge osteotomy, but this procedure is complex and meant only for simple malunions.

Constantine et al. (24) reviewed a total of 87,169 patients who underwent surgical fixation of distal radius fractures and found a significant difference in the rates of postoperative infection between open reduction and internal fixation and percutaneous pinning. Moreover, there was a 4-fold increase in the cost of care as a result of this infection. Malige et al. (25) compared 124 diabetic and 371 non-diabetic distal radius fractures and found a statistically higher rate of total complications (21% vs. 14%, *P* = 0.045), which is not affected by surgical timing.

Evans et al. (26) found that DDRMs resulted in a fundamental change in the biomechanics of the wrist. Untreated DDRMs may lead to arthritis, pain, limited mobility, or dysfunction. Decreased radial inclination results in a change in the direction of the flexor tendons, decreased mechanical advantage, and grip weakness (27). Loss of palmar tilt may lead to incongruity of the distal radioulnar joint, decreased forearm rotation, and wrist instability (28). Palmer et al. (29) suggested that radial shortening may lead to a shift of force transfer from the radiocarpal to the ulnocarpal joint, as much as a 42% increase. The optimal timing for surgery is still debated. Jupiter et al. (30) suggested that early (<14 weeks after injury) or late (≥14 weeks after injury) surgery are comparable.

Sato et al. (31) presented a series of 28 patients who underwent osteotomy for the treatment of volar DDRMs. In all patients, an iliac crest structural graft was used, followed by volar plating. All wrists united at the osteotomy site at an average of over 7 weeks, and supination improved to 80° from an average 16° preoperatively. McQueen et al. (32) treated 23 DDRMs by distal radial osteotomy using non-bridging external fixation and bone grafting. The mean preoperative dorsal angle of 20° was corrected to over 5° of volar tilt, and the mean preoperative positive ulnar variance of 3.9 mm was corrected to 2.5 mm. Brown et al. (33) treated 11 DDRMs with functional disability in the wrist due to limited wrist motion. Surgery consisted of opening wedge osteotomies to correct radial tilt and radial angle. Postoperatively, combined dorsal and volar flexion improved from a mean of 34° to 72°. Combined pronation and supination improved from a mean of 68° to 106°. Ma et al. (34) reviewed 68 patients with extra-articular DDRMs. They performed distal radius lengthening osteotomy in 32 patients and ulnar shortening osteotomy in 36 patients. They found that distal radius lengthening osteotomy achieved better reduction of pain and improvement of function. Ulnar shortening osteotomy is a simpler procedure but increases the risk of subsequent osteoarthritis of the distal radioulnar joint (25).

In the treatment of DDRMs at the distal levels, percutaneous distraction is a commonly used maneuver to restore the normal length of the radius (35). However, the distraction is compromised due to soft tissue contracture and elasticity of the ligament and capsule (27). Our bony distraction more effectively avoids these drawbacks. In this important procedure, the surgeon should palpate the radial artery and insert the pin 0.5 cm on the ulnar side away from the artery to avoid injuries to the artery and median nerve. DDRMs are often associated with poor bone quality, leading to osteoporosis (36). Bridging-external fixation can reduce axial load onto the distal fragments (37). Actually, radial height collapse is associated with poor wrist function, which mostly occurs in patients older than 65 years (38). In our study, patients were much younger, and therefore, the improved radiographic parameters may not translate into better functional scores in a shorter period of time.

The advantages of our technique are that it is minimally invasive with minimal tendon and wound complications. Fixation is reliable to maintain reduction, especially radial height. The procedure is simple because structure bone grafts are not required, thereby avoiding graft loosening, migration, and absorption. A single surgery reduces additional treatment cost and off work time because of implant removal and long periods of rehabilitation.

The disadvantage of our technique is pin site infection, but the incidence is low. Moreover, immobilization of the wrist may produce joint stiffness but no scar formation because the intactness of the capsule and surrounding ligaments may cause only minor morbidity to the wrist (39). The indications for our technique are extra-articular DDRMs, such as original AO/OTA Classification type A2 and A3 fractures and type C1 and C2 fractures, and even C3 fractures without the need to correct intraarticular malunions. In type B2 and B3 fractures, volar plating can provide rigid volar support, which is a contraindication of our technique.

This study has limitations. We did not perform any matching or adjustment to control confounders because the study was designed as a cohort study, and the relationship between the matching factors themselves and the outcomes were analyzed. Once matched, the variables are forced to be balanced between groups, and their independent effects can no longer be evaluated. We did not perform multivariate analyses for age, osteoporosis, or fracture type because the sample size of some categories was too small to support model complexity. The kinematics of fixation should require further study. The comparison groups belonged to different periods, which may result in temporal and selection bias and change in surgeon learning curve effects. Surgeon preference, experience, and abilities may influence wire configuration and placement. This study had a retrospective, non-randomized design, which increases the risk of selection bias based on fracture complexity or surgeon preference. Surgeons' experience improved over time, which may influence determination of the technical effects.

## Conclusions

DDRMs at distal levels can be treated via a 5 mm incision with external fixation through a small incision. External fixation through this small incision may be effective in maintaining radial height, especially in patients with osteoporosis. Usually, owing to a non-necessity for secondary implant removal in some patients, the technique results in acceptable outcomes in total treatment cost, total off work time, and patient satisfaction. The technique can be an alternative in addition to plate fixation. However, more factors should be considered when selecting the treatment strategy.

## Data Availability

The raw data supporting the conclusions of this article will be made available by the authors, without undue reservation.
